# The Association of Sagittal Spinal Posture among Elementary School Pupils with Sex and Grade

**DOI:** 10.3390/children11040446

**Published:** 2024-04-08

**Authors:** Stefan Đorđević, Mima Stanković, Bojan Jorgić, Saša Milenković, Semrija Smailović, Borko Katanić, Igor Jelaska, Luka Pezelj

**Affiliations:** 1 Faculty of Sport and Physical Education, University of Niš, 18000 Niš, Serbia; 2 Department of Pedagogical and Psychological Sciences, University of Novi Pazar, 36300 Novi Pazar, Serbia; 3 Montenegrin Sports Academy, 81110 Podgorica, Montenegro; 4 Faculty of Kinesiology, University of Split, 21000 Split, Croatia; jelaska@kifst.hr; 5 Faculty of Maritime Studies, University of Split, 21000 Split, Croatia

**Keywords:** adolescent, diagnosis, kyphosis, lordosis, posture, spinal curvatures

## Abstract

The objective of this research was to analyze and elucidate the sagittal spinal posture status in older elementary school children, considering their gender and grade differences. The study involved 484 school children (252 males and 232 females) from grades V to VIII, assessed for sagittal spinal posture using the Formetric 4D System. The analysis, employing the Chi-squared test of independence along with the Z-test, did not reveal significant grade-related differences (*p* < 0.52) in the incidence of normal sagittal alignment or diagnosed outliers. However, within grade levels, no significant difference was observed for male participants (*p* < 0.80), while a significant difference was identified for females (*p* < 0.01). Examining gender differences across grades, a disparity was noted only among seventh graders concerning normal spine alignment and outlier existence (*p* < 0.01), favoring male participants. Regardless of the grade, a significant gender difference emerged in the location of diagnosed outliers: kyphosis (M = 108 vs. F = 72), lordosis (M = 5 vs. F = 14), kypholordosis (M = 18 vs. F = 66), and flatback outlier of the lumbar spine (M = 27 vs. F = 11). These findings suggest potential adjustments to the curriculum and highlight the need to tailor physical education instruction based on this study’s outcomes. Consequently, these results imply the importance of a differentiated approach in preventing sagittal plane outliers of the spine in adolescent children.

## 1. Introduction

Proper postural status refers to the specific and relevant positioning of body segments at rest or during activity [1]. Children’s postural status is particularly vulnerable to significant negative impacts, especially during adolescence, as highlighted in numerous studies [2,3,4]. Inadequate posture and muscle imbalances can result in musculoskeletal pain and elevate the risk of injuries [5]. Therefore, timely detection and intervention in the postural status of adolescents are crucial. Research findings consistently point to a notably high prevalence of postural outliers during this age [6,7]. Furthermore, recent studies show a continuous rise in the prevalence of postural outliers, reported as a percentage [8]. The trend of an increased occurrence of poor posture among adolescents in the Republic of Serbia is also evident in neighboring countries within the Western Balkans region [9,10], as well as globally [6,11].

Sagittal spinal curves play a significant role as geometric factors influencing the mechanical characteristics and load balance on the intervertebral disc [12,13]. Postural deviations, particularly in the lumbar part of the sagittal plane, have been linked to scoliosis. Specifically, a flatback posture can serve as a triggering factor for the development of scoliosis in adolescents. Similarly, when examining the thoracic part of the sagittal plane of the spinal column with regard to kyphosis, this too is associated with scoliosis deformity and may serve as a predictor for its development [14].

Moreover, limited research has specifically addressed gender differences in the occurrence of postural outliers among school children [15,16]. Scarce studies have investigated the prevalence of postural outliers based on age or grade (fifth through eighth grade of elementary school), considering both the total sample size and gender. 

The primary limitations of the aforementioned studies pertain to inconsistent diagnostic methods, the use of measuring instruments with insufficient quality, and a restricted number of analyzed parameters. Furthermore, issues arise as all postural outliers are confined to the thoracic and lumbar sections of the sagittal plane of the spinal column. Additionally, questions remain regarding the most prevalent deformity in the sagittal plane of the spine and whether certain deformities are more prevalent in male or female respondents. Therefore, there is a need for a comprehensive posture analysis that covers a significant number of segments in the sagittal plane. Accordingly, this study aims to identify differences in sagittal plane outliers of the spine among older elementary school children of both genders, as well as variations among children in different grades.

## 2. Materials and Methods

### 2.1. Participants

The participant sample consisted of 484 school children attending fifth through eighth grades from the area of Knjaževac, Republic of Serbia (NM = 252 and NF = 232). A detailed description of the sample, as well as sub-samples by gender and grade, can be found in Table 1 Participants were included in the research if they were without any physical or mental disorders, aged 10 to 14 years. The participation of students was voluntary, and the research process adhered to the principles outlined in the Helsinki Declaration, with obtained written consent from parents. All participants were informed that they could leave the study without any penalties. The study was approved by the Ethics Committee of the University of Niš under no. 04-542/2.

### 2.2. Measures

To assess basic anthropometric parameters (body height, body mass, and BMI), a standardized anthropometric instrument (SECA model 284; SECA, Hamburg, Germany) was employed. Measurements using this instrument were conducted with the subjects wearing minimal clothing and no shoes, standing barefoot on the instrument stand in a clearly marked foot field and correct position. Results were digitally displayed after the measurement. The sagittal spinal posture of participants was measured using the Formetric 4D System (Diers, Schlangenbad, Germany), belonging to a group of non-invasive methods for diagnosing postural status, previously utilized in research [17,18,19]. The diagnostic method relied on photometry, based on the principle of triangulation. The system included a light projector projecting a line grid on the patient’s back, recorded by an imaging unit. Computer software analyzed the line curvature, generating a three-dimensional model of the surface through the Photogrammetry method, comparable to a plaster cast. In contrast to X-rays, the DIERS Formetric provides comprehensive information about the entire body statics and posture in a single measuring process, including spine curvature (lateral and frontal), vertebral rotation, and pelvic position. The instrument’s validity and reliability (ICC > 0.95) were determined in previous studies [20,21]. The works mentioned show a high correlation between rasterography and radiography (0.582). The software analysis provided values in degrees of angle for each analyzed segment. In Table 2, based on studies [22,23,24], the threshold values are shown, according to which deviations from normal spine values in the sagittal plane were determined. Reference values are provided according to the age of children.

### 2.3. Statistical Analysis

The data collected in this study were analyzed using SPSS 20 (SPSS Inc., Chicago, IL, USA). All data were presented through frequencies and percentages. To assess differences between genders and among different grades concerning the prevalence of spinal posture parameters, the Chi-square (χ^2^) test of independence was employed, along with the addition of the *Z*-test. Statistical significance was determined as *p* < 0.05 for all analyses.

## 3. Results

Table 3 presents the results for the total sample size (both genders), indicating the presence of sagittal plane outliers in 334 participants (69%). Among male participants, this figure was 170 (67.5%), while among female participants, sagittal plane outliers of the spine were more prevalent, with 164 participants (70.7%). Analyzing the total sample size based on grade revealed a consistent percentage increase in sagittal plane outliers of the spine (V = 100 (65.8%); VI = 76 (66.7%); VII = 85 (72.6%); VIII = 73 (72.3%)).

The findings presented in Table 4 indicate that, among older primary school children, the most frequently observed deviation in the sagittal plane of the spine is kyphosis (37.2%), followed by kypholordosis (17.4%), flatback in the lumbar section of the spine (7.9%), and lordosis (3.9%). Additionally, the results suggest that kyphosis is more prevalent in male participants (42.9% for males compared to 31% for females) and reaches its highest representation in the eighth grade (55.2%). Kypholordosis is more common among female participants (28.4% for females compared to 7.1% for males) and peaks in the eighth grade (41.9%). The postural issue of flatback in the lumbar section of the sagittal plane is more prevalent in male participants (10.7% for males compared to 4.7% for females) and reaches its highest point in the fifth grade (17.9%). Lordosis as a postural deviation is more frequently observed in female respondents (6% for females compared to 2% for males) and reaches its peak in the seventh grade (8.9%).

Based on Table 5, the results clearly indicate that. in the total sample size of participants, there was a significant difference in the prevalence of postural outliers (*p* < 0.01). Furthermore, the association between the typical postural status and deviations from the normal postural status in the sagittal plane of the spine differed in significance among classes within the overall sample (*p* = 0.520). However, this relationship was not statistically significant for male participants (*p* = 0.806), whereas it demonstrated statistical significance for female participants (*p* = 0.027).

The results describing sagittal plane outliers of the spine among older female elementary school children revealed a significant difference in the presence of postural outliers and normal alignment (*p* < 0.01). 

A more precise analysis of the results for each sagittal plane deformity of the spine among the grades indicated significant differences between the fifth and eighth grades, specifically for the flatback outlier deformity of the lumbar spine (V = 18; VI = 9; VII = 8; VIII = 3), for kypholordosis where significant differences were noted between all the grades and sixth grade (V = 25; VI = 14; VII = 26; VIII = 19), and the variables for kyphosis and the flatback outlier deformity of the lumbar spine as a sagittal imbalance between the fifth and sixth, seventh and eighth grades (V = 0; VI = 4; VII = 4; VIII = 4). A more detailed analysis of each sagittal plane outlier deformity indicated a significant difference between the analyzed grades, especially between the fifth and sixth, and seventh and eighth grades for the flatback outlier deformity of the lumbar spine (V = 14; VI = 3; VII = 7; VIII = 3), and the variables for kyphosis and the flatback outlier deformity of the lumbar spine as a sagittal imbalance between the fifth and sixth, and seventh and eighth grades (V = 0; VI = 4; VII = 4; VIII = 4). The results describing sagittal plane outlier deformities of the spine among older female elementary school children revealed a significant difference in the presence of postural outlier deformities and normal alignment (*p* < 0.01). A more detailed analysis of each sagittal plane outlier deformity indicated significant differences between the sixth and eighth grades for the flatback outlier deformity of the lumbar spine (V = 4; VI = 6; VII = 1; VIII = 0) and the variable for kypholordosis, where significant differences were noted between all grades and sixth grade (V = 16; VI = 11; VII = 21; VIII = 18).

Table 6 provides a more in-depth analysis of the results, specifically focusing on the difference in the prevalence of participants with normal sagittal alignment and those diagnosed with an outlier, based on grade. It only revealed a significant difference in the seventh grade, favoring male participants (normal alignment, male participants (23), female participants (9); deviations from normal alignment, male participants (38), female participants (47)).

In the results for the total sample size concerning gender differences in the deviation from normal sagittal alignment among older elementary school children, a significant difference is evident for the variable kyphosis, which is notably more prevalent among male participants (108) compared to female participants (72). Additionally, variable lordosis is more common among female participants (14) compared to male participants (5). The variable flatback outlier deformity of the lumbar spine, representing a sagittal imbalance, is also more prevalent among male participants (27) compared to female participants (11). Furthermore, the variable kypholordosis is more prevalent among female participants (66) compared to male participants (18).

## 4. Discussion

Proper postural status refers to the specific and relevant positioning of body segments at rest or during activity [1]. Children’s postural status is particularly vulnerable to significant negative impacts, especially during adolescence, as highlighted in numerous studies [2,3,4]. Aligned with the defined aims of this study and the processed data, the analysis of the results from the total sample size of participants revealed a highly significant difference between older elementary school children without an outlier and those with a sagittal plane outlier of the spine. Previous studies have consistently shown a high prevalence of sagittal plane outliers in this age group, and the current study indicates a trend of increasing percentages of postural outliers compared to prior research [25]. When comparing results from various studies [8,26], it is evident that the ages between 10 and 15 represent a period of intense change in sagittal spinal posture among participants, as corroborated by the findings in this study.

The observed high prevalence of postural outliers was not documented among younger elementary school children [8,27]. Similarly, studies involving slightly older participants did not report such high rates [28,29,30]. Therefore, the analyzed information suggests that the pre-adolescent period and early adolescence [31] are particularly susceptible to the emergence of postural outliers. These periods coincide with more intense growth and development of the human body, influenced not only by internal developmental changes but also by external environmental factors [32].

A more detailed analysis of the differences in the prevalence of normal alignment and sagittal plane outliers of the spine indicated significant differences in every grade, as well as in the total sample size of the participants.

When comparing these results with findings from researchers studying participants of the same age [6,7], significant differences can also be observed, but in favor of participants with an abnormal physiological curvature in sagittal spinal posture. An explanation for these differences may lie in the objective nature of the diagnosis method used in this study, as opposed to the subjective type employed in the cited studies. Analyzing the results based on gender for the total sample size of male participants reveals very high significant differences between those with normal alignment and those with sagittal plane outliers of the spine. The results regarding differences based on grade were not consistently significant; specifically, among male participants attending the seventh grade, no significant difference was noted. However, among female participants, a significant difference was observed in each grade, as well as for the total sample size of female participants. Comparing these findings with results from previous studies [7], a significant increase in sagittal plane outliers of the spine among seventh and eighth-grade girls, in comparison to younger elementary school children, was noted. These results, indicating varying levels of increase in postural outliers from the fifth to the eighth grade among boys and girls, can be attributed to diverse changes in biological development, which are physically manifested in alterations in the parameters of body composition, as confirmed in prior studies [33].

A more detailed analysis (Table 7) of the results for each individual sagittal plane outlier of the spine in the total sample size of participants based on grade, in general, did not indicate a significant difference in their prevalence. The exception is the lack of a normal physiological curvature in the lumbar spine among participants attending the fifth and eighth grades, where a significant difference was noted. Previous studies did not include results based on grade for this postural outlier but, based on these data, it can be observed that this outlier decreases with age. Additionally, at this age, significant differences emerge in the prevalence of kypholordosis among various grades. Analyzing previous research results also revealed an increase in this postural outlier among older elementary school children [8,27,34]. These results can be explained by the increasingly greater lack of physical activity among children at this age, as well as the emergence of compensatory postural outliers, primarily resulting from primary kyphosis and scoliosis. When analyzing the results (Table 7) for each individual sagittal plane outlier of the spine among male participants based on grade, in general significant difference was noted. The exception is a lack of normal physiological curvature in the lumbar spine among participants attending the fifth and eighth grades, where a significant difference can be found. Analyzing the results of previous studies [27,34], a decrease in the prevalence of this postural outlier can also be noted, even though it was previously examined in a general sense for both the thoracic and lumbar spine, and not for each segment individually, as was the case in this study. These results can be associated with an increase in body muscle mass and lean body mass, as well as a greater prevalence of organized physical activity among male participants at this age [35]. By analyzing the results for each individual sagittal plane outlier of the spine among the female participants based on grade, in general no significant difference can be noted. A more detailed analysis determined a significant difference between the female participants attending the fifth and sixth grades and those attending the seventh and eighth grades for kypholordosis. Analyzing the results of previous studies [27,34], an increase in the prevalence of this postural outlier among female elementary school children is evident, along with a higher percentage of occurrence compared to male participants of the same age. These results can be associated with an increase in body mass in the body composition and the trend of a decreasing prevalence of organized physical activity among female participants [36,37].

The results indicated that a significant gender difference between normal alignment and postural outliers was only noted in the seventh grade, while it was not noted for the remaining grades (Table 6). By analyzing the results of previous studies, which focused on the percentage of sagittal plane outliers of the spine based on each age group in elementary school, differences can be noted in the prevalence of postural outliers between boys and girls at the age of approximately 13 [6,7]. By analyzing the results pertaining to the differences in the prevalence of sagittal plane outliers of the spine among older elementary school children for the total sample size, significant differences between the two genders can be noted in general. By analyzing gender differences based on grade, a significant difference was not found only for the fifth grade (Table 8). The most prevalent outlier among children is kyphosis, and it is significantly more prevalent among the male participants. However, by analyzing the differences among grades, a significant difference was noted between the sixth and eighth grades, while no differences between the genders was noted for the fifth and seventh grades. In previous studies [27,34], kyphosis stood out as the most prevalent sagittal plane outlier of the spine, and its prevalence was statistically greater among the male population of participants, in general and by grades (Table 8). The second most prevalent outlier is kypholordosis, which is significantly more prevalent among the female participants. Among younger elementary school children, there was a significantly lower prevalence of this postural outlier [8,27] while, among participants who are older than those in the analyzed sample, this postural outlier is also significantly prevalent compared to the remaining sagittal plane outliers of the spine [26]. The results can be accounted for by the biological development primarily of the female participants, who have a more inclined pelvis, but it is also reflected in the transformation of both the internal and external features of femininity in the thoracic region, which are characteristic of this phase of biological development. The third most prevalent outlier is the flatback outlier of the lumbar spine which is significantly more prevalent among the male participants. This result can be a consequence of phylogenetic development, considering that the lumbar curvature is the final segment of the spine to form. Lordosis, as the fourth most prevalent sagittal plane outlier of the lumbar spine, has a significant higher prevalence among the female participants. This postural outlier is significantly more prevalent among female participants in all the studied grades (Table 8), but also in general in the results of previous studies, where the presence of this postural outlier was diagnosed more frequently among female participants [38]. The reason for the increased presence of this postural outlier among female participants is explained by the biological positioning of the pelvis, as well as a proportionally reduced length of the femur [39].

This study distinguishes itself through a thorough analysis of a large population of adolescents, employing exceptionally precise and contemporary measuring instruments. Of particular significance is the inclusion of a substantial number of segments in the sagittal plane, a feature not previously addressed in research involving participants from the territory of the Republic of Serbia [27]. A gender difference in the prevalence of outliers located in the thoracic and lumbar parts of the sagittal spine was observed. Moreover, gender differences in specific outliers and the degree of their presence in the thoracic and lumbar parts of the sagittal plane of the spine were also determined. The information gathered in this manner underscores the importance of preventive measures, particularly in elementary schools where physical and health education instructors can play a crucial role, such as in this study, because the subjects who exhibited postural deviations in the sagittal plane of the spinal column were provided with exercise program recommendations tailored to address their specific postural deviations. It also emphasizes the need for developing a prevention program that takes into account gender and age characteristics. Adopting such an approach to address the issue of sagittal plane postural status in children, especially during periods of higher prevalence, can contribute to maintaining a normal posture and halting the progression of existing deformities. This strategy has the potential to reduce the occurrence of health issues associated with these deformities, such as back pain, limited mobility in the thoracic and lumbar areas, and impacts on the function of respiratory organs and heart rate. Ultimately, such an approach could lead to a decrease in the need for costly and risky surgical interventions.

A limitation of the study is that it did not include the cervical and sacral parts of the spinal column in the sagittal plane. Additionally, the study did not specify how many children reported to have outliers in the sagittal plane of the spinal column in the thoracic and lumbar regions also have scoliosis. A recommendation for future research would be to analyze the postural status of children’s spinal column in the sagittal plane, including the cervical and sacral parts. Moreover, conducting a comprehensive analysis of the postural status of children in younger grades in elementary school would contribute to gaining a more complete insight into the postural status of children in the sagittal plane of the spinal column.

## 5. Conclusions

The results of this study affirm a high prevalence of sagittal plane outliers of the spine among elementary school children. However, it was determined, based on the results, that there is a continued tendency for a percentage increase among children according to the grade they attend in elementary school, as well as gender differences in the prevalence of spinal outliers. Male participants exhibit a lower prevalence of sagittal plane outliers of the spine compared to female participants. Furthermore, it can be concluded, based on the results, that there are differences in the prevalence of outliers in the seventh and eighth grades, favoring male participants. A more detailed analysis revealed a significant gender difference in the location of the diagnosed outlier, indicating that kyphosis and the flatback deformity of the lumbar spine are more prevalent among male participants, while lordosis and kyphosis are more present among female participants. These findings underscore the necessity of a different approach in the prevention of sagittal plane outliers of the spine among adolescent children.

## Figures and Tables

**Table 1:** Description of the sample of participants.

			Body Height (Mean ± SD)	Body Mass (Mean ± SD)	BMI (Mean ± SD)
M (252)			155.90 ± 11.14	51.43 ± 15.96	20.84 ± 4.82
F (232)			153.95 ± 8.75	49.19 ± 12.65	20.54 ± 4.22
T (484)			154.96 ± 10.11	50.36 ± 14.50	20.69 ± 4.54
**G**	**Group (*n*)**	**Age**			
V	M (78)	10.62 ± 0.53	147.26 ± 6.88	43.33 ± 13.08	19.77 ± 4.94
	F (74)	10.68 ± 0.49	147.29 ± 8.12	43.69 ± 12.25	19.90 ± 4.33
	T (152)	10.65 ± 0.51	147.28 ± 7.49	43.51 ± 12.64	19.83 ± 4.64
VI	M (55)	11.74 ± 0.44	153.52 ± 9.05	49.17 ± 13.05	20.68 ± 4.18
	F (59)	11.83 ± 0.49	153.81 ± 6.75	48.54 ± 13.31	20.32 ± 4.55
	T (114)	11.78 ± 0.47	153.67 ± 7.91	48.84 ± 13.13	20.49 ± 4.36
VII	M (61)	12.66 ± 0.51	159.98 ± 10.13	56.76 ± 17.11	21.86 ± 4.86
	F (56)	12.78 ± 0.49	153.67 ± 6.73	53.65 ± 11.84	21.42 ± 4.19
	T (117)	12.71 ± 0.49	158.88 ± 8.71	55.27 ± 14.84	21.65 ± 4.54
VIII	M (58)	13.67 ± 0.47	165.48 ± 8.87	58.86 ± 15.48	21.35 ± 4.98
	F (43)	13.76 ± 0.42	160.74 ± 6.37	53.77 ± 9.47	20.79 ± 3.42
	T (101)	13.71 ± 0.45	163.46 ± 8.21	56.69 ± 13.44	21.11 ± 4.38

Legend: G—grade; M—male; F—female; T—total.

**Table 2:** Variables for the evaluation of spinal posture.

No.	Variable	Abbreviation
1.	Normal sagittal alignment of the spine involves an angle of convexity in the thoracic section ranging from 20 to 45° and an angle of concavity in the lumbar section ranging from 30 to 48° (for individuals aged 11–13). For those aged 14 to 16 years, the angle in the lumbar part is from 53°. Kyphosis is identified by an angle in the thoracic part of the spinal column in the sagittal plane where the convexity exceeds 45°.	NorSagAl
2.		Kyph
3.	Flatback outlier of the thoracic spine (the angle in the thoracic part of the sagittal plane of the spinal column is less than 20°)	FltTho
4.	Lordosis (from 11 to 13 years old with a concavity greater than 48° and for subjects aged 14 to 16 years greater than 53°)	Lord
5.	Flatback outlier of the lumbar spine (from 11 to 13 years old with a concavity of less than 30°)	Fltlym
6.	Kypholordosis (when the subject exhibits an angle of convexity in the thoracic part greater than 45° and a concavity in the lumbar part greater than 48° (11–13 years old) or 53° (14–16 years old))	Kyphlor
7.	Kyphosis and flatback outlier of the lumbar spine/sagittal imbalance(when the subject has an angle of convexity in the thoracic part greater than 45° and concavity in the lumbar part less than 30°)	KyfFlaLymSag
8.	Outlier from normal sagittal alignment of the spine	DevNormSagAl

**Table 3:** Postural status of the sagittal plane of the spine—frequency and percentage.

			NorSagAl	DevNormSagAl
G	P	N	(#/%)	(#/%)
V	M	78	24/30.8	54/69.2
	F	74	28/37.8	46/62.2
	T	152	52/34.2	100/65.8
VI	M	55	17/30.9	38/69.1
	F	59	21/35.6	38/64.4
	T	114	38/33.3	76/66.7
VII	M	61	23/37.7	38/62.3
	F	56	9/16.1	47/83.9
	T	117	32/27.4	85/72.6
VIII	M	58	18/31.	40/69.0
	F	43	10/23.3	33/76.7
	T	101	28/27.7	73/72.3
Total	M	252	82/32.5	170/67.5
	F	232	68/29.3	164/70.7
	T	484	150/31.0	334/69.0

Legend: G—grade; M—male; F—female; T—total; P—gender; NorSagAl—normal sagittal alignment of the spine; DevNormSagAl—deviation from normal sagittal alignment of the spine.

**Table 4:** Postural deviations in the sagittal plane of the spinal column—frequency and percentage.

			Kyph	FltTho	Lord	Fltlym	Kyphlor	Kyfflalymsags	NorSagAl
G	P	N	(#/%)	(#/%)	(#/%)	(#/%)	(#/%)	(#/%)	(#/%)
V	M	78	28/35.9		3/3.8	14/17.9	9/11.5		24/30.8
	F	74	21/28.4	1/1.4	4/5.4	4/5.4	16/21.4		28/37.8
	T	152	49/32.2	1/0.7	7/4.6	18/11.8	25/16.4		52/34.2
VI	M	55	27/49.1		1/1.8	3/5.5	3/5.5	4/7.3	17/30.9
	F	59	18/30.5		3/5.1	6/10.2	11/18.1		21/35.6
	T	114	45/39.5		4/3.5	9/7.9	14/12.3	4/3.5	38/33.3
VII	M	61	21/34.4		1/1.6	7/11.5	5/8.2	4/6.6	23/37.7
	F	56	20/35.7		5/8.9	1/1.8	21/37.5		9/16.1
	T	117	41/35		6/5.1	8/6.8	26/22.2	4/3.4	32/27.4
VIII	M	58	32/55.2			3/5.2	1/1.7	4/6.9	18/31.
	F	43	13/30.2		2/4.7		18/41.9		10/23.3
	T	101	45/44.6		2/2.0	3/3.0	19/18.8	4/4.0	28/27.7
Total	M	252	108/42.9		5/2.0	27/10.7	18/7.1	12/4.8	82/32.5
	F	232	72/31.0	1/0.4	14/6.0	11/4.7	66/28.4		68/29.3
	T	484	180/37.2	1/0.2	19/3.9	38/7.9	84/17.4	12/2.5	150/31.0

Legend: G—grade; M—male; F—female; T—total; P—gender; Kyph—kyphosis; FltTho—flatback outlier of the thoracic spine; Lord—lordosis; Fltlym—flatback outlier of the lumbar spine; Kyphlor—kypholordosis; Kyfflalymsags—kyphosis and flatback outlier of the lumbar spine/sagittal imbalance.

**Table 5:** The difference in the postural status of the spinal column in the sagittal plane in students, older grades in elementary school.

	G	NorSagAl	DevNormSagAl	*Z* Test Sig	χ^2^ Sig
T (484)	V	52 ^a^	100 ^a^	0.000	0.000
	VI	38 ^a^	76 ^a^	0.000	
	VII	32 ^a^	85 ^a^	0.000	
	VIII	28 ^a^	73 ^a^	0.000	
		χ^2^ + *Z* test		0.520	
M (252)	V	24 ^a^	54 ^a^	0.001	**0.000**
	VI	17 ^a^	38 ^a^	0.006	
	VII	23 ^a^	38 ^a^	0.072	
	VIII	18 ^a^	40 ^a^	0.005	
		χ^2^ + *Z* test		0.806	
F (232)	V	28 ^a^	46 ^a^	0.047	**0.000**
	VI	21 ^a^	38 ^a^	0.036	
	VII	9 ^b^	47 ^b^	0.000	
	VIII	10 ^a,b^	33 ^a,b^	0.000	
		χ^2^ + *Z* test		**0.027**	

Legend: G—grade; M—male; F—female; T—total; NorSagAl—normal sagittal alignment of the spine; DevNormSagAl—deviation from normal sagittal alignment of the spine; χ^2^—level of significance of the Chi-square test of independence; *Z* test sig—significance of the difference; χ^2^ + *Z* test—significance of the match in the differences in postural status in the sagittal plane; ^a^, ^b^—each subscript letter denotes a subset of sex categories whose column proportions do not differ significantly from each other at the 0.05 level.

**Table 6:** The disparity in the sagittal plane postural status of the spinal column among older grade elementary school students of different genders.

G	P	NorSagAl	DevNormSagAl	χ^2^ + *Z* Test
V	M	24 ^a^	54 ^a^	0.359
	F	28 ^a^	46 ^a^	
VI	M	17 ^a^	38 ^a^	0.596
	F	21 ^a^	38 ^a^	
VII	M	23 ^a^	38 ^a^	0.009
	F	9 ^b^	47 ^b^	
VIII	M	18 ^a^	40 ^a^	0.388
	F	10 ^a^	33 ^a^	
T	M	82 ^a^	170 ^a^	0.443
	F	68 ^a^	164 ^a^	

Legend: G—grade; M—male; F—female; T—total; P—gender; NorSagAl—normal sagittal alignment of the spine; DevNormSagAl—deviation from normal sagittal alignment of the spine; χ^2^ + *Z* test—significance of the match in the differences in postural status in the sagittal plane; ^a^, ^b^—each subscript letter denotes a subset of sex categories whose column proportions do not differ significantly from each other at the 0.05 level.

**Table 7:** The variation in postural deviations within the sagittal plane of the spinal column among older grade elementary school students.

	G	Kyph	FltTho	Lord	Fltlym	Kyphlor	Kyfflalymsags	χ^2^ Sig
T (484)	V	49 ^a^	1 ^a^	7 ^a^	18 ^a^	25 ^a^	0 ^a^	0.126
	VI	45 ^a^	0 ^a^	4 ^a^	9 ^a,b^	14 ^b^	4 ^b^	
	VII	41 ^a^	0 ^a^	6 ^a^	8 ^a,b^	26 ^a^	4 ^b^	
	VIII	45 ^a,b^	0 ^a^	2 ^a^	3 ^b^	19 ^a^	4 ^a^	
M (252)	V	28 ^a^		3 ^a^	14 ^a^	9 ^a^	0 ^a^	0.025
	VI	27 ^a,b^		1 ^a^	3 ^b^	3 ^a,b^	4 ^b^	
	VII	21 ^a^		1 ^a^	7 ^a,b^	5 ^a,b^	4 ^b^	
	VIII	32 ^b^		0 ^a^	3 ^b^	1 ^b^	4 ^b^	
F (232)	V	21 ^a^	1 ^a^	4 ^a^	4 ^a,b^	16 ^a,b^		0.218
	VI	18 ^a^	0 ^a^	3 ^a^	6 ^b^	11 ^b^		
	VII	20 ^a^	0 ^a^	5 ^a^	1 ^a,b^	21 ^a,b^		
	VIII	13 ^a^	0 ^a^	2 ^a^	0 ^a^	18 ^a^		

Legend: G—grade; M—male; F—female; T—total; Kyph—kyphosis; FltTho—flatback outlier of the thoracic spine; Lord—lordosis; Fltlym—flatback outlier of the lumbar spine; Kyphlor—kypholordosis; Kyfflalymsags—kyphosis and flatback outlier of the lumbar spine/sagittal imbalance; χ^2^ sig—level of significance of the Chi-square test of independence; ^a^, ^b^—each subscript letter denotes a subset of sex categories whose column proportions do not differ significantly from each other at the 0.05 level.

**Table 8:** The variations in postural deviations within the sagittal plane of the spinal column among students of different genders and grades in elementary school.

G	P	Kyph	FltTho	Lord	Fltlym	Kyphlor	Kyfflalymsags	χ^2^ Sig
V	M	28 ^a^	0 ^a^	3 ^a^	14 ^a^	9 ^a^		0.059
	F	21 ^a^	1 ^a^	4 ^a^	4 ^b^	16 ^a^		
VI	M	27 ^a^		1 ^a^	3 ^a^	3 ^a^	4 ^a^	0.015
	F	18 ^b^		3 ^a^	6 ^a^	11 ^b^	0 ^b^	
VII	M	21 ^a^		1 ^a^	7 ^a^	5 ^a^	4 ^a^	0.000
	F	20 ^a^		5 ^a^	1 ^b^	21 ^b^	0 ^a^	
VIII	M	32 ^a^		0 ^a^	3 ^a^	1 ^a^	4 ^a^	0.000
	F	13 ^b^		2 ^a^	0 ^a^	18 ^b^	0 ^a^	
T	M	108 ^a^	0 ^a^	5 ^a^	27 ^a^	18 ^a^	12 ^a^	0.000
	F	72 ^b^	1 ^a^	14 ^b^	11 ^b^	66 ^b^	0 ^b^	

Legend: G—grade; M—male; F—female; T—total; P—gender; Kyph—kyphosis; FltTho—flatback outlier of the thoracic spine; Lord—lordosis; Fltlym—flatback outlier of the lumbar spine; Kyphlor—kypholordosis; Kyfflalymsags—kyphosis and flatback outlier of the lumbar spine/sagittal imbalance; χ^2^—level of significance of the Chi-square test of independence; ^a^, ^b^—each subscript letter denotes a subset of sex categories, whose column proportions do not differ significantly from each other at the 0.05 level.

## Data Availability

The original contributions presented in the study are included in the article, further inquiries can be directed to the corresponding author.
